# Evidence of a species complex within the food-borne trematode *Opisthorchis viverrini* and possible co-evolution with their first intermediate hosts

**DOI:** 10.1016/j.ijpara.2006.12.008

**Published:** 2007-05

**Authors:** Weerachai Saijuntha, Paiboon Sithithaworn, Sopit Wongkham, Thewarach Laha, Vichit Pipitgool, Smarn Tesana, Neil B. Chilton, Trevor N. Petney, Ross H. Andrews

**Affiliations:** a Department of Biochemistry, Faculty of Medicine, Khon Kaen University, Khon Kaen 40002, Thailand; b Department of Parasitology, Faculty of Medicine, Khon Kaen University, Khon Kaen 40002, Thailand; c Liver Fluke and Cholangiocarcinoma Research Center (LFCRC), Faculty of Medicine, Khon Kaen University, Khon Kaen 40002, Thailand; d Department of Biology, University of Saskatchewan, Saskatoon, Sask., Canada S7N 5E2; e School of Pharmacy and Medical Sciences, University of South Australia, GPO Box 2471, Adelaide, SA 5001, Australia; f Institute of Zoology 1: Ecology and Parasitology, University of Karlsruhe, Kornblumen Strasse 13, Karlsruhe, Germany

**Keywords:** *Opisthorchis viverrini*, Food-borne trematodes, Species complex, Co-evolution, Multilocus enzyme electrophoresis, *Bithynia*, Snails, Intermediate host

## Abstract

The food-borne trematodes, *Opisthorchis viverrini*, *O. felineus* and *Clonorchis sinensis*, have long been recognized as the cause of major human health problems, with an estimated 40 million infected persons. Of the three species of liver fluke, only *O. viverrini* is classified as a type 1 carcinogen because of its role as an initiator of chronic inflammation and the subsequent development of cholangiocarcinoma. At present, there are no techniques for the early diagnosis of cholangiocarcinoma and it is fatal for most patients. There is considerable variation in parasite prevalence and disease presentation in different geographical areas, the latter of which may be associated with genetic differences among parasites. In the present study, multilocus enzyme electrophoresis was used to provide a comprehensive genetic characterization of *O. viverrini* from different geographical localities in Thailand and the Peoples’ Democratic Republic of Laos. Parasites from different localities were compared genetically at 32 enzyme loci. The results of the genetic analyses are sufficient to reject the null hypothesis that *O. viverrini* represents a single species. Therefore, *O. viverrini* consists of at least two genetically distinct, yet morphologically similar (i.e. cryptic) species. Moreover, there was also separation of the different populations of snails (i.e. the first intermediate hosts) into two distinct genetic groups that corresponded with the delineation of *O. viverrini* into two species. This suggests that there may be a history of co-evolution in this host–parasite lineage. Additionally, five distinct genetic groups of parasites were detected, each of which occurred within a different and independent river wetland system. Our findings have major implications for the implementation of effective control and surveillance programs targeted to these medically important food-borne parasites.

## 1 Introduction

The food-borne trematode *Opisthorchis viverrini* occurs in Thailand, the Peoples’ Democratic Republic of Laos (Laos PDR), Cambodia and Vietnam (WHO, 1995). It has a three-host life cycle. The first intermediate hosts are snails of three subspecies, *Bithynia siamensis siamensis*, *B. siamensis goniomphalos* and *B. funiculata* (see Kaewkes, 2003). At least 18 species of cyprinid fish act as the second intermediate host, whereas carnivores (e.g., cats and dogs), but more specifically humans, are definitive hosts (WHO, 1995). Humans become infected with this parasite following the consumption of raw or partially cooked cyprinid fish containing metacercariae. Infection in humans causes hepatobiliary disease with the subsequent development, in most cases, of cholangiocarcinoma (CHCA) (IARC, 1994; Sithithaworn et al., 1994; Honjo et al., 2005).

This liver fluke is endemic in Southeast Asia, with an estimated nine million people infected, predominately in Thailand (Sithithaworn and Haswell-Elkins, 2003). In the highest endemic region of northeast Thailand, it has been estimated that opisthorchiasis costs US $120 million annually in lost wages and medical care (Muller, 2002). Its prevalence in snails is generally low (<1%), whereas in cyprinid fish prevalence is extremely high (90–95%) (Vichasri et al., 1982; Brockelman et al., 1986). Additionally, there are significant differences in the prevalence and intensity of infection by *O. viverrini* in humans between geographical areas of Thailand (Jongsuksuntigul et al., 1992; Jongsuksuntigul and Imsomboon, 1998). Endemic areas of opisthorchiasis in Thailand are mainly in the northeast and north but the parasite is absent in the south (Jongsuksuntigul and Imsomboon, 2003). The prevalence of *O. viverrini* is at its highest in the northeast, but the rate of infection in humans varies considerably among villages, districts and provinces (Sithithaworn and Haswell-Elkins, 2003). For instance, prevalence in the northeast was reported to range from an average of 14% in Khon Kaen Province to 34% in Nakhon Phanom Province (Jongsuksuntigul, 2002), some 300 km apart. However, a more recent report (Sriamporn et al., 2004) indicates that *O. viverrini* infection ranges from 2% to 71% (mean 25%) in different districts within Khon Kaen Province. The prevalence of *O. viverrini* infection in the neighboring country of Laos PDR has been estimated to be as high as 60% (Sithithaworn and Haswell-Elkins, 2003). There is also variation in the incidence of CHCA in different communities in Thailand, however, there is no apparent correlation with the prevalence of *O. viverrini* (see Sriamporn et al., 2004). To date, the role of genetic variation of *O. viverrini* on this variability in prevalence, transmission and associated disease is not known.

Multilocus enzyme electrophoresis (MEE) has been used very effectively over many years to study the population genetics and systematics of a variety of parasitic helminths (Andrews and Chilton, 1999). However, this technique has not been applied to examine the magnitude of genetic variation in *O. viverrini* from different geographical regions. The aim of the present study was to use MEE to provide significant new insights on genetic variability of this parasite of socio-economic importance. In addition, genetic variation among parasites from different river wetland systems was compared with the genetic variation of their first intermediate hosts (snails) collected from the same geographic localities.

## 2 Materials and methods

### 2.1 Collection of adult *O. viverrini* and *Bithynia* snails

**Fig. 1:**
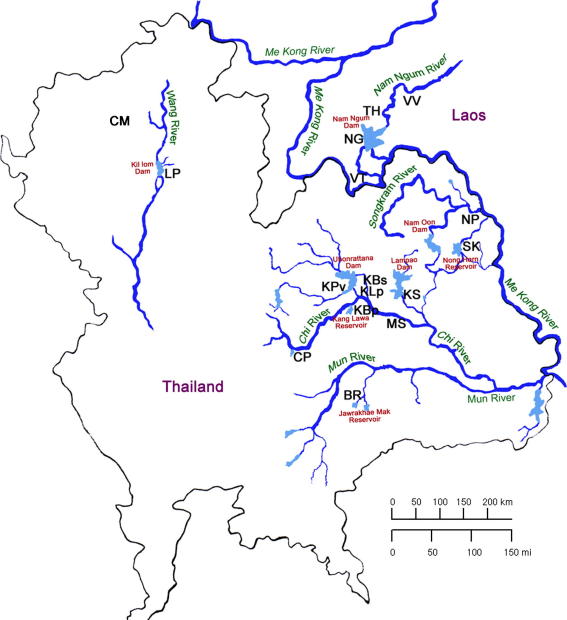
Locations in Thailand and the Peoples’ Democratic Republic of Laos (Laos PDR) from where trematodes and/or snails were collected. The abbreviations of the localities (geographical source; Province or village) are listed in Table 1.

Metacercariae of *O. viverrini* were obtained from infected cyprinid fish collected from five different major river wetland systems, including dams, natural reservoirs and secondary branches, in Thailand (11 localities) and Laos PDR (four localities) (Table 1 and Fig. 1). To ensure a high recovery of metacercariae, several hundred fish from each collection locality were pooled and their combined tissues (≈weight of 1–10 kg) digested with 0.3% pepsin solution. Pooled fish samples were used because infected fish contain a small number of metacercariae. It was also important to combine metacercariae from a large number of fish from each locality for the electrophoretic analyses because single fish may contain clones of a single fluke and therefore would not provide a reliable indication of the genetic diversity of *O. viverrini* at that locality. Metacercariae were identified using a light microscope, 50–100 metacercariae were used to infect hamsters, with at least five hamsters used per collection locality. After 4–6 months, adult worms were collected from the bile duct, identified using morphological characters (Kaewkes, 2003), then washed extensively in physiological saline, placed in pools (⩾20 worms), into microcentrifuge tubes, frozen live and stored at −80 °C for subsequent electrophoretic analyses. Pools of worms were used to provide sufficient sample volume to enable comprehensive electrophoretic analyses at the largest number of loci possible. Although the approach of using pools of individuals, rather than screening large numbers of single individuals, has some disadvantages (i.e. unable to determine the frequency of different alleles in a population), one advantage is that alleles occurring at a low frequency in the population are more likely to be detected in pools of 10 or more individuals rather than in a smaller number of individuals (Chilton et al., 1993).

Snails (*B. s. goniomphalos*) were also collected for MEE from most localities from which *O. viverrini* metacercariae were obtained. The one exception was that a different species of snail (*B. funiculata*) was collected from Chiang Mai, in the Mae Ping River wetland (Table 1 and Fig. 1).

The project protocol was been approved by the Animal Ethics committee, Faculty of Medicine, Khon Kaen University. Procedures of care and use of animals recommended in the guidelines of the National Ethical Committee on animal experimentation, National Research Council of Thailand, are strictly followed.

### 2.2 Multilocus enzyme electrophoresis (MEE)

Frozen homogenates of *O. viverrini* and uninfected hamster liver (i.e. the host enzyme control on gels) were prepared for electrophoretic examination by adding an equal volume of lysing solution (100 ml distilled H_2_O, 100 μl β-mercaptoethanol, 10 mg nicotinamide adenine dinucleotide phosphate) to the thawed sample, sonicating and centrifuging at 8000*g* for 10 min at 4 °C. Supernatants were stored in capillary tubes as about 5 μl aliquots at −20 °C until used. MEE was conducted on a pool of worms from each geographical area and host liver tissue, using cellulose acetate (Cellogel; Milan) as the support medium. Each gel was stained histochemically for a specific enzyme (Richardson et al., 1986). Of the 46 enzymes examined, 28 enzymes encoding a presumptive 32 loci gave sufficient staining intensity and resolution to enable reliable genetic interpretation of pooled samples of at least 20 *O. viverrini* (Table 2). The 28 enzymes used and their Enzyme Commission (E.C.) numbers were: aconitate dehydrogenase (ACON; E.C. 4.2.1.3), adenylate kinase (AK, E.C. 2.7.4.3), aldolase (ALD, E.C. 4.1.2.13), creatine kinase (CK, E.C. 2.7.3.2), enolase (ENOL, E.C. 4.2.1.11), esterase (EST, E.C. 3.1.1.1), fructose-1,6-diphosphatase (FDP, E.C. 3.1.3.11), fumarate hydratase (FUM, E.C. 4.2.1.2), glyceraldehyde-3-phosphate dehydrogenase (GAPD, E.C. 1.2.1.12), glucose-6-phosphate dehydrogenase (G6PD, E.C. 1.1.1.49), aspartate amino transferase (GOT, E.C. 2.6.1.1), glucose-phosphate isomerase (GPI, E.C. 5.3.1.9), alanine amino transferase (GPT, E.C. 2.6.1.2), hexokinase (HK, E.C. 2.7.1.1), isocitrate dehydrogenase (IDH, E.C. 1.1.1.42), lactate dehydrogenase (LDH, E.C. 1.1.1.27), malate dehydrogenase (MDH, E.C. 1.1.1.37), malic enzyme (ME, E.C. 1.1.1.40), nucleotide diphosphate kinase (NDPK, E.C. 2.7.4.6), peptidase valine–leucine (PEPA, E.C. 3.4.13.11), peptidase leucine–glycine–glycine (PEPB, E.C. 3.4.11.4), peptidase phenylalanine–proline (PEPD, E.C. 3.4.13), phosphoglycerate mutase (PGAM, E.C. 2.7.5.3), phosphoglucomuatase (PGM, E.C. 2.7.5.1), 6-phosphogluconate dehydrogenase (6PGD, E.C. 1.1.1.44), pyruvate kinase (PK, E.C. 2.7.1.40), triose phosphate isomerase (TPI, E.C. 5.3.1.1) and uridine monophosphate kinase (UMPK, E.C. 2.7.1.48). Five individual snails from each locality were also subjected to MEE using the same methodology as for the parasites. Each snail was genetically characterized at 19 enzymes encoding a presumptive 26 loci (Table 3). Two of the enzymes used to compare genetic variation among snails, glycerol-3-phosphate dehydrogenase (αGPD, E.C. 1.1.1.8) and purine nucleoside phosphorylase (NP, E.C. 2.4.2.1), were not used in the comparison of the parasites.

### 2.3 Interpretation of MEE banding patterns and data analyses

For each locus, the electrophoretic banding pattern of each sample was interpreted allozymically, that is, the allozyme with the least electrophoretic mobility from the cathode was designated as allele *a*. The multiple banding patterns of individual snails for two enzyme loci (*Ak* and ∝*Gpd*) were consistent with the expectations of heterozygous individuals for enzymes with either a monomeric (AK) or dimeric (∝GPD) quaternary structure. As a consequence of using pools of individual *O. viverrini* to maximize the number of available genetic markers for analysis, a number of isolates exhibited multiple bands at nine enzyme loci (*Acon*, *Ak*, *Enol*, *Gapd*, *Hk*, *Mdh*, *Ndpk*, *Pgm* and *Tpi*). The banding patterns were often consistent with the expectations of heterozygous individuals within the pools of adult worms for enzymes with either a monomeric (ACON and PGM) or dimeric (ENOL, MDH and TPI) quaternary structure. However, this will be confirmed in future studies by examining the banding patterns of individual worms for these loci. Nonetheless, the presence of multiple bands in isolates does not affect interpretation of the data with respect to the proportion of loci with ‘fixed’ allelic/genetic differences between samples (i.e. where an isolate does not share any alleles in common with another isolate at a particular locus) (Richardson et al., 1986). This genetic distance measure is believed to be appropriate for applications of MEE to studies of the systematics of organisms and has been shown to correlate with other genetic distance measures, such as Nei’s D (Richardson et al., 1986).

Phenograms were constructed using Unweighed Pair–Group Method with Arithmetic averages (UPGMA) (Sneath and Sokal, 1978) analysis of pair-wise comparisons of the proportion of loci that showed fixed allelic differences between *O. viverrini* isolates or between snails from different localities.

## 3 Results

Based on our previous MEE studies to define a sufficient number of enzyme loci to genetically characterise *O. viverrini* (Saijuntha et al., 2006a,b) 28 enzymes encoding a presumptive 32 loci were used herein for the genetic characterization of *O. viverrini*. This number of enzyme loci is considerably more than that employed in MEE studies on other species of food-borne trematodes (Agatsuma et al., 1986; 1994; Park et al., 2002) and provides the opportunity to assess the significance of genetic variation detected among geographical localities, and to define a sufficient number of independent genetic markers for biological and epidemiological studies.

Comparison of the allelic profiles of *O. viverrini* (Table 2) revealed no differences among isolates at 13 loci. At seven of these (*Fum-1*, *Gpt-2*, *Pep-a*, *Pep-b*, *Pep-d*, *Pgam* and *Umpk*) all *O. viverrini* isolates had a different single-banded pattern each (i.e. allozyme, hence allele) compared with that of the related food-borne trematode, *Fasciola gigantica* (Table 2). These thus represent useful genetic markers to distinguish the two species. These loci may represent potential genetic markers to distinguish *O. viverrini* from other sympatric species of food-borne trematode (e.g., *Clonorchis sinensis*) that are morphologically similar to *O. viverrini* and/or produce similar clinical effects (Honjo et al., 2005). Allelic variation among *O. viverrini* isolates was detected at 19 (60%) enzyme loci (Tables 2 and 4). Some of these polymorphic loci can be used to distinguish *O. viverrini* from different geographical areas. For example, all *O. viverrini* from Thailand possess different alleles to *O. viverrini* originating in Laos PDR for the enzyme loci *Ck* and *Enol*. In addition, *O. viverrini* from Khon Kaen Province (Thailand) have different alleles from *O. viverrini* in Laos PDR at a further nine enzyme loci (*Acon, Ck, Enol, Est, Got-1, Gpi, Gpt-1, Me-2* and *Pk*), and can be distinguished from *O. viverrini* from the other provinces in Thailand using a single enzyme locus (*Got-1*). Fixed genetic differences, where samples do not share any alleles at a specific locus (Richardson et al., 1986; Andrews and Chilton, 1999), were also detected among isolates from Khon Kaen Province at three loci: *Ald, G6pd* and *Pk* (Table 2).

The extent of the fixed differences among the four *O. viverrini* isolates from Khon Kaen Province (3–9%) was equivalent to that among the four isolates from Laos PDR (3–9%), but significantly less than that among isolates from different provinces in Thailand (3–41%; mean = 22%) or between countries (i.e. Thailand vs Laos PDR: 17–44%; mean = 30%) (Table 4).

**Fig. 2:**
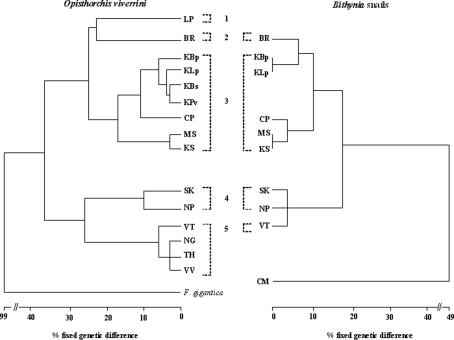
Comparison of phenograms depicting percent fixed genetic differences between isolates of *Opisthorchis viverrini* from different localities in five different wetlands (1, Wang River; 2, Mun River; 3, Chi River; 4, Songkram River; 5, Nam Ngum River) and fixed genetic differences between the snail host, *Bithynia siamensis goniomphalos*, from the same localities.

The phenogram (Fig. 2) of the MEE data (Tables 2 and 4) shows the delineation of *O. viverrini* into two major groups, each representing at least one species with fixed differences at >30% of loci, with further subdivision into two to six clusters, each of which is distinguished from others by fixed genetic differences of ⩾15%. The first and second cluster are represented by a single isolate from Lampang (LP) and Buri Ram (BR), respectively (22%). The third cluster contained five isolates, four from Khon Kaen Province (KBp, KLp, KBs and KPv) and one isolate from Chaiya Phum Province (CP). The fourth cluster contained two isolates from Mahasarakham Province (MS) and Kalasin Province (KS), which had 17% fixed genetic differences from the third cluster. The fifth contained two isolates from Sakon Nakhon Province (SK) and Nakhon Phanom Province (NP), which differed from the sixth cluster containing four isolates from Laos (VT, NG, TH and VV) at 25% fixed genetic differences.

Fixed genetic differences at 17% of loci were detected between *B. s. goniomphalos* from the Songkram River + Nam Ngum River wetlands and the Chi River + Mun River wetlands (Tables 3 and 5). This separation of the intermediate hosts into two major genetic groups corresponded with the major division detected among isolates of *O. viverrini*.

## 4 Discussion

For sexually reproducing diploid organisms, as is the case for *O. viverrini*, fixed genetic differences among two sympatric populations at a single locus would indicate the lack of gene flow (Richardson et al., 1986; Andrews and Chilton, 1999), and hence establish the existence of two species, while two or more fixed genetic differences provide unequivocal evidence of two sympatric species. However, in comparisons of allopatric populations, which was the case in the present study, fixed genetic differences at >15% of enzyme loci are generally indicative of the existence of distinct species, whereas fixed differences of <15% reflect genetic variation among populations of a single species (Richardson et al., 1986; Andrews and Chilton, 1999). Nonetheless, it is prudent to obtain other evidence (e.g., biological, morphological, ecological) to confirm the specific status of geographically isolated populations. This being the case, we have adopted a highly conservative interpretation of the MEE data by using ⩾30% fixed genetic differences as an indicator of species delineation (i.e. twice that used in other studies to distinguish species complexes of geographically isolated populations (Richardson et al., 1986; Andrews and Chilton, 1999). Therefore, the magnitude of the fixed differences detected between isolates of *O. viverrini* from some geographical localities is sufficient to reject the null hypothesis that it represents a single species. Specifically, *O. viverrini* from the localities of Vang Vieng (VV), Nam Ngum (NG), Tha Heur (TH) and Vientiane (VT) in Laos PDR are all genetically distinct (with fixed differences at 33–44% of loci) from, and thus represent a different species to, *O. viverrini* from the Thai provinces of Khon Kaen (KBs, KLp, KBp and KPv), Chaiya Phum (CP), Mahasarakham (MS) and Kalasin (KS). The conclusion that *O. viverrini* does not represent a single species is also supported by independent molecular and biological evidence.

Preliminary investigations on the fecundity of *O. viverrini*, based on the number of eggs/gm/worm following infection of metacercariae in hamsters, have also revealed significant differences between *O. viverrini* from TH (Laos PDR) and CP (Thailand) (Sithithaworn et al., unpublished). In addition, the results of random amplification of polymorphic DNA (RAPD) analyses on *O. viverrini* from some of the localities used in the present study, revealed that individuals from Nam Ngum Province (Laos PDR) were genetically distinct from those from the Thai provinces of Chaiya Phum, Mahasarakham, Kalasin and Khon Kaen (which includes the localities of KBs, KLp, KBp and Nampong) (Sithithaworn et al., 2007). This finding is in agreement with the results of the MEE data.

Our study has revealed another finding of major biological and epidemiological significance, namely, the separation of *O. viverrini* into distinct genetic groups/clusters that are associated with different wetlands. The significance of the genetic divergence within clusters (i.e. within different areas of some wetlands) from a taxonomic perspective is unclear. Whether these genetic differences are indicative of population/geographical variation within a single species, or evidence of additional cryptic species, remains to be determined following comprehensive MEE analysis of multiple isolates from each wetland and surrounding geographical areas and additional biological data. Nonetheless, of major significance is the detection of considerable genetic variation among *O. viverrini* from different geographical areas. This now provides the basis to determine, we believe for the first time, whether parasite genetic variation is associated with disease presentation that has been recorded in different geographical areas.

Comparisons of the *O. viverrini* phenogram with that of its first intermediate host, *B. s. goniomphalos* (Fig. 2) revealed major similarities in the patterns of genetic divergence of both parasites and snails. *Bithynia s. goniomphalos* from both the Songkram River and Nam Ngum River wetlands were genetically distinct (fixed differences at 17% of loci) from individuals from the Chi River and Mun River wetlands. This separation of the *B. s. goniomphalos* into two major genetic groups was in total concordance with the delineation of *O. viverrini* into two species. MEE analyses were conducted on *B. s. goniomphalos* because this subspecies of snail occurs in Laos PDR and throughout northeast Thailand (Wykoff et al., 1965; Brockelman et al., 1986), which included most of the localities from where *O. viverrini* were collected in our study. One exception was Kil Lom Dam (LP; Lampang Province) on the Wang River wetland, where *B. s. goniomphalos* is replaced by *B. funiculata* as the first intermediate host. Specimens of *B. funiculata* from a neighboring province, Chiang Mai (CM) (Fig. 1), were used in the analyses (Fig. 2). The third subspecies of snail used by *O. viverrini*, *B. s. siamensis*, only occurs in central Thailand (Upatham and Sukhapanth, 1980).

Our findings raise interesting questions concerning the possibility of co-evolution between species of *O. viverrini* and their first intermediate hosts, and the role of wetland systems as isolating mechanisms preventing gene flow between parasite populations. This three-host parasite system may therefore represent an ideal model to examine fundamental questions relating to the evolution of parasites and their hosts. More detailed analyses, using the genetic markers established in the present study, are required to determine the extent of associations between specific genotypes of *O. viverrini* and snails, as well as the second intermediate hosts (fish), in different wetlands. This will provide a sound foundation within which to better understand the origin, occurrence, pattern and spread of opisthorchiasis through human populations and to examine, in detail, whether correlations exist concerning the incidence of CHCA, and the presence of specific *O. viverrini* genotype(s) that initiate the disease.

Given the magnitude of the genetic variation detected among *O. viverrini* from different wetlands and among *B. s. goniomphalos* from different geographical areas, and the possibility that different genotypes/species of *O. viverrini* may be associated with different rates of opisthorchiasis in humans, further work will be undertaken to determine whether *O. viverrini* can establish successful infections in snails from different geographical areas. Furthermore, the relative importance of the biotic and abiotic features of different wetlands on the ecology and genetic variation of trematodes and their intermediate hosts also needs to be explored. Such studies are of critical importance given the potential for the establishment of mixed species/genotypes as a consequence of an increase in relocation of persons to different regions within Southeast Asia (e.g., Laotian refugees and laborers relocated to Thailand), some of whom will be infected by adults worms of a different parasite genotype/species. This may also occur in years of extensive flooding of different wetlands. Such scenarios have major implications for the surveillance, control and treatment regimes for opisthorchiasis.

In conclusion, analyses of the MEE data demonstrate that distinct genetic populations and/or species of *O. viverrini* sensu lato are associated with different major river wetland systems, which include Chi River, Mun River, Songkram River and the Wang River in Thailand, and the Nam Ngum River in Laos PDR. The separation of the parasite into at least two sibling species is supported by preliminary evidence from other molecular (Sithithaworn et al., 2007) and biological data (Sithithaworn et al., unpublished) and coincides with the division of *B. s. goniomphalos* into two major genetic groups. Detection of a new species of food-borne parasite may provide an explanation for the considerable variation in the currently available epidemiological data (e.g. parasite prevalence, intensity, morbidity data, etc.) relating to *O. viverrini*. Given that the human populations in these wetlands are potentially infected with different species and/or distinct genetic populations of *O. viverrini*, it is now necessary to determine what these differences are and whether control strategies and treatment (medication) will be equally effective between areas in Southeast Asia, where an estimated 67.3 million people are at risk of infection with *O. viverrini* (see Keiser and Utzinger, 2005).

## Figures and Tables

**Table 1:** Details of the source of the 15 isolates of *Opisthorchis viverrini* and/or of the *Bithynia* snails used in this study

Code	Collecting locality	Wetland	Province (village/district)	Country
KBs	Kang Namton Reservoir	Chi River	Khon Kaen (Ban Sa-ard)	Thailand
KLp	Prakeu Stream	Chi River	Khon Kaen (Ban Lerngpleuy)	Thailand
KBp	Kang Lawa Reservoir	Chi River	Khon Kaen (Ban Phai)	Thailand
KPv	Ubonrattana Dam	Chi River	Khon Kaen (Phuviang)	Thailand
CP	Nong Ben Reservoir	Chi River	Chaiya Phum	Thailand
MS	Chi River	Chi River	Mahasarakham	Thailand
KS	Lampao Dam	Chi River	Kalasin	Thailand
LP	Kil Lom Dam	Wang River	Lampang	Thailand
CM	Rice field near Mae Ping River	Mae Ping River	Chiang Mai (Mae Rim)	Thailand
BR	Huay Jawrakhae Mak Reservoir	Mun River	Buri Ram	Thailand
SK	Nong Harn Reservoir	Songkram River	Sakon Nakhon	Thailand
NP	Songkram River	Songkram River	Nakon Phanom	Thailand
VV	Nam Ngum Dam	Nam Ngum River	Vang Vieng	Laos PDR
NG	Nam Ngum Dam	Nam Ngum River	Nam Ngum	Laos PDR
TH	Nam Ngum Dam	Nam Ngum River	Tha Heur	Laos PDR
VT	Nam Ngum Dam	Nam Ngum River	Vientiane	Laos PDR

**Table 2:** Alleles (*a*–*f*) detected at 32 enzyme loci among isolates of *Opisthorchis viverrini* collected from different geographical localities

Enzyme locus	Geographical localities^a^															
	KBs	KLp	KBp	KPv	CP	MS	KS	LP	BR	SK	NP	VV	NG	TH	VT	Fg^b^
*Acon*	*c*,*d*	*c*,*d*	*c*,*d*	*c*,*d*	*c*,*d*	*c*,*d*	*c*,*d*	*e*,*f*	*e*,*f*	*e*,*f*	*e*,*f*	*e*,*f*	*e*,*f*	*e*,*f*	*e*,*f*	*a*,*b*
*Ak*	*c*,*d*	*c*,*d*	*c*,*d*	*c*,*d*	*c*,*d*	*c*,*d*	*c*,*d*	*c*,*d*	*c*,*d*	*c*,*d*	*c*,*d*	*c*,*d*	*c*,*d*	*c*,*d*	*c*,*d*	*a*,*b*
*Ald*	*b*	*b*	*c*	*b*	*b*	*c*	*c*	*c*	*b*	*c*	*c*	*c*	*c*	*c*	*c*	*a*
*Ck*	*a*	*a*	*a*	*a*	*a*	*a*	*a*	*a*	*a*	*a*	*a*	*b*	*b*	*b*	*b*	*c*
*Enol*	*b*,*d*	*b*,*d*	*b*,*d*	*b*,*d*	*b*,*d*	*b*,*d*	*b*,*d*	*b*,*d*	*b*,*d*	*b*,*d*	*b*,*d*	*a*,*c*	*a*,*c*	*a*,*c*	*a*,*c*	*e*
*Est*	*b*	*b*	*b*	*b*	*b*	*b*	*b*	*b*	*b*	*a*	*a*	*a*	*a*	*a*	*a*	*c*
*Fd p*	*a*	*a*	*a*	*a*	*a*	*a*	*b*	*b*	*b*	*a*	*a*	*a*	*b*	*b*	*a*	*c*
*Fum-1*	*a*	*a*	*a*	*a*	*a*	*a*	*a*	*a*	*a*	*a*	*a*	*a*	*a*	*a*	*a*	*b*
*Fum-2*	*b*	*b*	*b*	*b*	*a*	*a*	*a*	*b*	*a*	*b*	*b*	–	*a*	*b*	*a*	–
*Gapd*	*c*,*d*	*c*,*d*	*c*,*d*	*c*,*d*	*e*,*f*	*e*,*f*	*e*,*f*	*e*,*f*	*e*,*f*	*c*,*d*	*c*,*d*	–	*c*,*d*	*c*,*d*	*c*,*d*	*a*,*b*
*G6pd*	*b*	*b*	*a*	*a*	*b*	*c*	*c*	*a*	*b*	*a*	*a*	*b*	*b*	*b*	*b*	*c*
*Got-1*	*b*	*b*	*b*	*b*	*a*	*a*	*a*	*a*	*a*	*a*	*a*	*a*	*a*	*a*	*c*	*d*
*Got-2*	*c*	*c*	*c*	*c*	*c*	*b*	*b*	*c*	*c*	*c*	*c*	*c*	*c*	*c*	*c*	*a*
*Gpi*	*b*	*b*	*b*	*b*	*b*	*c*	*c*	*c*	*a*	*c*	*c*	*a*	*a*	*a*	*a*	*d*
*Gpt-1*	*b*	*b*	*b*	*b*	*b*	*b*	*b*	*b*	*c*	*b*	*c*	*c*	*c*	*c*	*c*	*a*
*Gpt-2*	*b*	*b*	*b*	*b*	*b*	*b*	*b*	*b*	*b*	*b*	*b*	*b*	*b*	*b*	*b*	*a*
*Hk*	*a*,*b*	*a*,*b*	*a*,*b*	*a*,*b*	*a*,*b*	*a*,*b*	*a*,*b*	*a*,*b*	*a*,*b*	*a*,*b*	*a*,*b*	*a*,*b*	*a*,*b*	*a*,*b*	*a*,*b*	*c*,*d*
*Idh*	*c*	*c*	*c*	*c*	*c*	*c*	*c*	*c*	*b*	*b*	*b*	*b*	*b*	*b*	*b*	*a*
*Ldh*	*c*	*c*	*c*	*c*	*c*	*c*	*c*	*c*	*c*	*b*	*b*	*c*	*c*	*c*	*c*	*a*
*Mdh*	*a*,*c*	*a*,*c*	*a*,*c*	*a*,*c*	*a*,*c*	*a*,*c*	*a*,*c*	*a*,*c*	*a*,*c*	*a*,*c*	*a*,*c*	*a*,*c*	*a*,*c*	*a*,*c*	*a*,*c*	*b*,*d*
*Me-1*	*b*	*b*	*b*	*b*	*b*	*c*	*c*	*c*	*c*	*c*	*b*	*b*	*b*	*b*	*b*	*a*
*Me-2*	*b*	*b*	*b*	*b*	*b*	*a*	*a*	*b*	*b*	–	*c*	*c*	*c*	*c*	*c*	–
*Ndpk*	*c*,*d*	*c*,*d*	*c*,*d*	*c*,*d*	*c*,*d*	*c*,*d*	*c*,*d*	*c*,*d*	*c*,*d*	*c*,*d*	*c*,*d*	*c*,*d*	*c*,*d*	*c*,*d*	*c*,*d*	*a*,*b*
*Pep-a*	*b*	*b*	*b*	*b*	*b*	*b*	*b*	*b*	*b*	*b*	*b*	*b*	*b*	*b*	*b*	*a*
*Pep-b*	*b*	*b*	*b*	*b*	*b*	*b*	*b*	*b*	*b*	*b*	*b*	*b*	*b*	*b*	*b*	*a*
*Pep-d*	*b*	*b*	*b*	*b*	*b*	*b*	*b*	*b*	*b*	*b*	*b*	*b*	*b*	*b*	*b*	*a*
*Pgam*	*b*	*b*	*b*	*b*	*b*	*b*	*b*	*b*	*b*	*b*	*b*	*b*	*b*	*b*	*b*	*a*
*6Pgd*	*a*	*a*	*a*	*a*	*b*	*b*	*a*	*b*	*b*	*a*	*a*	*a*	*a*	*a*	*a*	*a*
*Pgm*	*b*,*c*	*b*,*c*	*b*,*c*	*b*,*c*	*b*,*c*	*b*,*c*	*b*,*c*	*b*,*c*	*b*,*c*	*b*,*c*	*b*,*c*	*b*,*c*	*b*,*c*	*b*,*c*	*b*,*c*	*a*
*Pk*	*b*	*c*	*b*	*b*	*c*	*b*	*b*	*c*	*c*	*b*	*d*	*d*	*d*	*d*	*d*	*a*
*Tpi*	*c*,*d*	*c*,*d*	*c*,*d*	*c*,*d*	*c*,*d*	*c*,*d*	*c*,*d*	*c*,*d*	*c*,*d*	*c*,*d*	*c*,*d*	*c*,*d*	*c*,*d*	*c*,*d*	*c*,*d*	*a*,*b*
*Umpk*	*b*	*b*	*b*	*b*	*b*	*b*	*b*	*b*	*b*	*b*	*b*	*b*	*b*	*b*	*b*	*a*

– Insufficient staining activity.

a Isolate codes listed in Table 1.

b *Fasciola gigantica*.

**Table 3:** Alleles (*a*–*g*) detected at 26 enzyme loci among snails, *Bithynia siamensis goniomphalos* from different geographical localities and *Bithynia funiculata* from Chiang Mai (CM)

Enzyme locus	Geographical localities^a^									
	KBp	KLp	CP	MS	KS	BR	SK	NP	VT	CM
*Ak-1*	*b*,*c*	*c*	*b*,*c*	*b*,*c*	*b*,*c*	*b*,*c*	*b*,*c*	*a*,*b*,*c*	*a*,*b*	*a*,*b*
*Ak*-2	*a*	*a*,*b*	*a*,*b*	*a*,*c*	*b*,*c*	*b*,*c*	*b*	*a*,*b*,*c*	*a*,*b*	*b*
*Ck*	*a*	–	*a*	*a*	*a*	*a*	*a*	*a*	*a*	*b*
*Enol*	*b*	*b*,*c*	*b*	*b*	*b*	*a*,*b*	*b*,*c*	*a*,*b*,*c*	*b*	*a*,*b*
*Est-1*	*b*,*c*	*b*,*c*,*d*	*b*	*b*,*d*	*b*,*e*	*b*,*d*,*e*	*c*	*b*,*c*	*c*,*d*	*a*,*b*
*Est-2*	*b*,*c*	*b*,*c*	*b*	*b*,*c*	*b*	*b*	*b*	*b*	*b*,*c*	*a*,*d*
*Fum*	*a*,*b*	*b*	*b*	*b*	*b*	*b*	*a*,*b*	*a*,*b*	*a*,*b*	*a*,*c*
*Gapd*	*c*,*d*	–	*c*	*c*	*b*,*c*,*d*	*c*	*a*,*b*,*c*	*b*,*c*	*c*	*a*,*b*,*c*
α*Gpd*	*b*,*c*,*d*	*d*,*e*	*d*	*d*,*e*	*c*,*d*,*e*,*f*	*b*,*c*,*d*,*e*	*b*,*d*	*a*,*b*	*a*,*b*	*a*,*b*
*G6pd*	*b*,*c*	*c*,*d*	*b*	*b*	*b*	*c*,*d*	*b*,*d*	*a*,*b*	*a*,*b*	*d*,*e*
*Got-1*	*c*	*c*	*b*	*b*,*c*	*b*,*c*	b	*a*,*b*	*a*,*b*	*b*	*a*,*b*
*Got-2*	*c*,*d*	*c*,*d*	*c*	*b*,*c*,*d*	*c*,*d*	*b*	*b*	*a*,*b*,*d*	*a*,*b*	*b*,*c*,*d*
*Gpt*	*b*,*c*	*b*	*b*	*b*	*b*	*b*	*a*,*b*	*a*,*b*	*a*,*b*	*a*,*b*
*Idh*	*a*	*a*,*b*	*a*,*b*	*a*,*b*	*b*	*b*	*b*	*a*,*b*	*b*	*a*,*b*
*Mdh*	*b*,*c*	*c*	*c*	*c*	*b*,*c*	*c*	*b*	*a*,*b*	*b*	*a*,*b*
*Me*	*c*	*c*	*b*	*b*	*b*	*c*	*c*	*c*	*d*	*a*,*b*
*Ndpk-1*	*b*,*c*	*b*,*c*	*d*,*e*	*c*	*b*,*c*	*b*,*c*,*d*	*a*,*b*	*a*,*b*,*c*	*b*,*c*,*e*	*b*,*c*
*Ndpk-2*	*b*	*b*,*c*	*b*	*b*	*b*,*c*	*b*,*c*	*c*	*a*,*b*	*a*,*b*	*b*
*Np-1*	*b*,*c*	*c*,*d*,*e*	*c*,*d*	*b*,*c*	*c*,*d*	*c*,*d*,*e*	*b*	*a*,*b*	*a*,*c*,*d*	*f*,*g*
*Np-2*	*b*	*b*	*b*	*b*	*b*	*b*	*b*	*b*	*b*	*a*
*Pgam-1*	*a*	*a*	*a*	*a*	*a*,*b*	*a*	*a*,*b*	*a*,*b*	*a*,*b*	*c*
*Pgam-2*	*a*	*a*	*a*	*a*	*a*	*a*	*a*,*b*	*a*	*a*	*b*
*6Pgd*	*b*,*c*	*b*,*c*	*b*,*c*	*b*,*c*	*c*	*c*	*b*,*c*	*b*,*c*	*b*,*c*	*a*
*Pk*	*b*	*b*	*b*	*b*	*b*	*b*	*b*	*b*	*b*	*a*
*Umpk-1*	*b*	*b*,*c*	*b*	*b*,*c*	*a*,*b*,*c*	*c*	*a*,*c*	*a*,*b*	*a*,*b*	*a*,*b*
*Umpk-2*	*b*	*b*	*b*	*a*,*b*	*a*,*b*	*b*	*b*	*a*,*b*	*b*	*b*

– Insufficient staining activity.

a Isolate codes listed in Table 1.

**Table 4:** Pairwise comparison of the percentage of fixed genetic difference among isolates *Opisthorchis viverrini* and *Fasciola gigantica* (Fg) collected from different geographical localities

Code^a^	KBs	KLp	KBp	KPv	CP	MS	KS	LP	BR	SK	NP	VV	NG	TH	VT	Fg
KBs	–															
KLp	3	–														
KBp	6	9	–													
KPv	3	6	3	–												
CP	9	6	16	13	–											
MS	16	19	13	16	13	–										
KS	19	22	16	19	16	3	–									
LP	25	22	19	22	22	25	22	–								
BR	25	22	31	28	16	28	25	22	–							
SK	32	35	26	29	35	32	35	19	35	–						
NP	38	38	31	34	38	38	41	28	38	10	–					
VV	37	37	37	40	33	37	40	37	30	28	17	–				
NG	41	41	41	44	34	38	34	34	25	32	22	3	–			
TH	38	38	38	41	38	41	38	31	28	29	19	3	3	–		
VT	38	38	38	41	34	38	41	41	31	32	22	3	6	9	–	
Fg	100	100	100	100	100	97	97	100	100	97	97	100	100	100	100	–

a Abbreviations from Table 1.

**Table 5:** Pairwise comparison of the percentage of fixed genetic difference among *Bithynia* snails from different geographical localities

Code^a^	KBp	KLp	CP	MS	KS	BR	SK	NP	VT	CM
KBp	–									
KLp	0	–								
CP	12	17	–							
MS	4	8	4	–						
KS	12	8	4	0	–					
BR	15	4	15	8	8	–				
SK	23	21	35	27	15	15	–			
NP	4	17	19	8	8	15	4	–		
VT	15	29	19	12	12	19	12	4	–	
CM	50	54	54	54	46	50	38	46	50	–

a Abbreviations from Table 1.
